# U. S. V. M. A.

**Published:** 1895-09

**Authors:** 


### SOCIETY MEETINGS AND PROCEEDINGS.

### U. S. V. M. A.

Under the head of Society Proceedings we publish a brief prospectus of the coming Missouri State meeting at Moberly on September 4th. Surely the programme should attract a large attendance, and preceding the coming national meeting in their midst, the election of a delegate, and a large representation at Des Moines, add additional importance.

Kansas City and vicinity will be well represented at Des Moines. We hear little from St. Louis, Mo., in regard to the national meeting. The Association has some very old and earnest members in that city, and they should all be present at this meeting in their midst. Distance will lend no excuse this year.

Professor S. J. J. Harger, of the Committee on Contagious Diseases, will summarize those which have been prevalent in Pennsylvania the past year, and to which will be added "Remarks Upon Recent English and Continental Investigations of Contagious Diseases," all of which will be interesting news to the members of the Association.

Chairman Butler, of the Prize Committee, desires to announce that all original papers submitted for the annual prize offered by the Association shall be sent addressed to him at Agricultural College, Mississippi; these papers to be sealed and marked in the usual manner.

Professors Gill, Huidekoper, and possibly Huddleston, of the New York Veterinary College, will be among the party out by the B. and O. that leaves Philadelphia at 7.55 A.M. on the morning of the 7th of September.

New York promises to be well represented at the meeting in Des Moines. The meeting of the State Association in New York on September 5th and 6th will give impetus to the interest and number present.

Alabama, Virginia, District of Columbia, Mississippi, Maryland, and Delaware will lead as representatives below Mason and Dixon's line.

Kansas, Missouri, Iowa, and Illinois will form a strong contingent numerically at the coming meeting.

Secretary Ramsey, of Missouri, is doing very earnest work among the veterinarians of that State, in view of the coming meeting of the Association at Des Moines.

Buffalo seems to have no competition for 1896. She deserves an unchallenged field.

Secretary Piatt, of the Missouri State Veterinary Association, is looking forward to the largest meeting of their organization that they have had, at Moberly, Mo., on September 4th, and this promises to inure to the benefit of the meeting at Des Moines.

Vice-President Turner says that Western veterinarians, or those beyond the Mississippi, look forward with a great deal of pride and pleasure to what they can consider really the first Western meeting of the U. S. V. M. A.

Treasurer Robertson, the faithful watchdog of the treasury, promises to be early on the grounds to direct his little boomlet for the Pacific Coast in 1897.

How many of the old guard of 1890 and 1893 will be on hand to renew the pleasures and social enjoyments to Chicago and the West, that made merry the hours and pleasant the memories of former journeys to our Conventions?

The Kiamensi quartette, under the leadership of Messrs. Faust and Foelker, will again conduct the musical festivities on the outward-bound passage to Des Moines.

Ex-Secretary Turner, in his official migrations, is doing yeoman work for the success of the meeting at Des Moines.

It is earnestly requested that all veterinarians from the East travelling to Des Moines will endeavor to be in Chicago on the afternoon of the 8th of September to join the party travelling by the B. and O. from Philadelphia to join in the reception and luncheon tendered by the McKillip Veterinary College.

Arrangements have been made whereby the tickets from Philadelphia, by special party over the Baltimore and Ohio, will be made good to return until the 20th of September.

If all our members were as active in missionary service for the success of the Des Moines meeting as Vice-President Turner we would have an awakening among the profession that would be a rare experience in our meetings.

Secretary Pearson announces the granting of the usual one and one-third rates by the Western, Southern, and Central Traffic Association for the delegates to the meeting at Des Moines.

It is expected that Dr. T. D. Hinebauch will again take up the subject of "Millet Disease" at Des Moines, a subject well considered by him at Chicago in 1893, and of the cause of which he had not arrived at definite conclusions.

The subject of "Professional Charges" will be considered in a paper by Professor R. P. Huidekoper at the 1895 meeting. A great deal of interest will be attached to this announcement, owing to the very exalted views of the worth of professional veterinary services entertained by the writer, in addition to the great importance of the subject in the present depressed and demoralized condition of animal industry.

